# Effectiveness of a Group-Based Culturally Tailored Lifestyle Intervention Program on Changes in Risk Factors for Type 2 Diabetes among Asian Indians in the United States

**DOI:** 10.1155/2017/2751980

**Published:** 2017-01-11

**Authors:** Rupal M. Patel, Ranjita Misra, Sudha Raj, Ashok Balasubramanyam

**Affiliations:** ^1^School of Physical Therapy, Texas Woman's University, 6700 Fannin Street, Houston, TX 77030, USA; ^2^Department of Social & Behavioral Sciences, Room No. 3313A, Robert C Byrd Health Science Center, School of Public Health, West Virginia University, Morgantown, WV 26506-9190, USA; ^3^Department of Public Health, Food Studies and Nutrition, The David B. Falk College of Sport and Human Dynamics, Syracuse University, Syracuse, NY, USA; ^4^Department of Medicine, Diabetes, Endocrinology and Metabolism, Baylor College of Medicine, Houston, TX, USA

## Abstract

This study used an experimental, pretest-posttest control group repeated measures design to evaluate the effectiveness of a community-based culturally appropriate lifestyle intervention program to reduce the risk for type 2 diabetes (T2DM) among* Gujarati* Asian Indians (AIs) in an urban community in the US. Participants included 70 adult AIs in the greater Houston metropolitan area. The primary outcomes were reduction in weight and hemoglobin A1c (HbA1c) and improvement in physical activity. Participants were screened for risk factors and randomly assigned to a 12-week group-based lifestyle intervention program (*n* = 34) or a control group (*n* = 36) that received standard print material on diabetes prevention. Participants also completed clinical measures and self-reported questionnaires about physical activity, social, and lifestyle habits at 0, 3, and 6 months. No significant baseline differences were noted between groups. While a significant decline in weight and increase in physical activity was observed in all participants, the intervention group lowered their HbA1c (*p* < 0.0005) and waist circumference (*p* = 0.04) significantly as compared to the control group. Findings demonstrated that participation in a culturally tailored, lifestyle intervention program in a community setting can effectively reduce weight, waist circumference, and HbA1c among* Gujarati* AIs living in the US.

## 1. Introduction

Globally, 415 million people have Type 2 Diabetes Mellitus (T2DM) with a projected increase to 642 million people by 2040 [1]. T2DM has escalated more rapidly in developing countries due to several factors: aging of the population, population growth, urbanization, increasing prevalence of obesity, and physical inactivity [2–5]. Currently, countries with the highest cases of diabetes are China, India, and the US [6]. In the United States, 29.1 million Americans have diabetes and 86 million have prediabetes [7]. Racial and ethnic minorities have higher prevalence of diabetes than Non-Hispanic Whites; Asian Indians (AIs), one of the fastest growing Asian subgroups, are disproportionally burdened by the disease [8].

An analysis of 3-year aggregate data from the National Health Interview Survey (NHIS) showed that Asian Indians, the largest South Asian group in the US, were 130% more likely to have diabetes [9]. A higher prevalence of T2DM among AI adults (14%–35%) has been reported in the literature as compared to other Asian subgroups and the general US population [7, 10, 11] due to several risk factors. Some of these are nonmodifiable (e.g., intrauterine malnutrition [12], genetic disposition [13, 14], ethnicity [15, 16], and age [10, 17, 18]) but several modifiable risk factors increase with acculturation and westernization [19]: overweight/obesity [16], abdominal adiposity [17], high blood pressure [20], physical inactivity [21], and poor diet [22–24]. Furthermore, US Asian Indians tend to be sedentary and have a higher prevalence of overweight/obesity than other Asian American subgroups [9, 25].

Lifestyle intervention has shown to be effective in preventing or delaying the onset of T2DM for at-risk multiethnic American [26], Finnish [27], Chinese [28], and Indian [29] populations. For example, in the US Diabetes Prevention Program (DPP) trial [26], a 58% reduction in diabetes risk was achieved through modest weight loss (5 to 7% of body weight) and 30 minutes of moderate-intensity exercise 5 times weekly. This lifestyle intervention was shown to be more effective then metformin in a 10-year follow-up study [30].

Translation of the DPP has taken place in various settings from primary care clinics [31], healthcare facilities [32, 33], and worksites [34, 35] to community centers [36]. Programs that have tailored the DPP materials to address language, cultural, and economic barriers have been effective in high risk Latino [37] and African American [38] populations. Yet, to date, few interventions have targeted AIs, the largest Asian subgroup in Texas [8] with a higher burden of T2DM than other racial/ethnic subgroups [10]. Studies investigating the effectiveness of culturally adapted DPP translational programs among AIs in the US are scarce [39, 40]. Also, AIs are diverse representing various religious beliefs, languages, and cultural customs.* Gujarati *Asian Indians, the largest subgroup of Asian Indians living in the US, are predominantly vegetarians with distinct lifestyle habits and risk factors [41–43]. Thus, the purpose of this study was to evaluate the effectiveness of a culturally tailored, community-based lifestyle intervention among at-risk* Gujarati* Asian Indians in an urban community in the US.

## 2. Methods

### 2.1. Research Design and Study Framework

**Figure 1:**
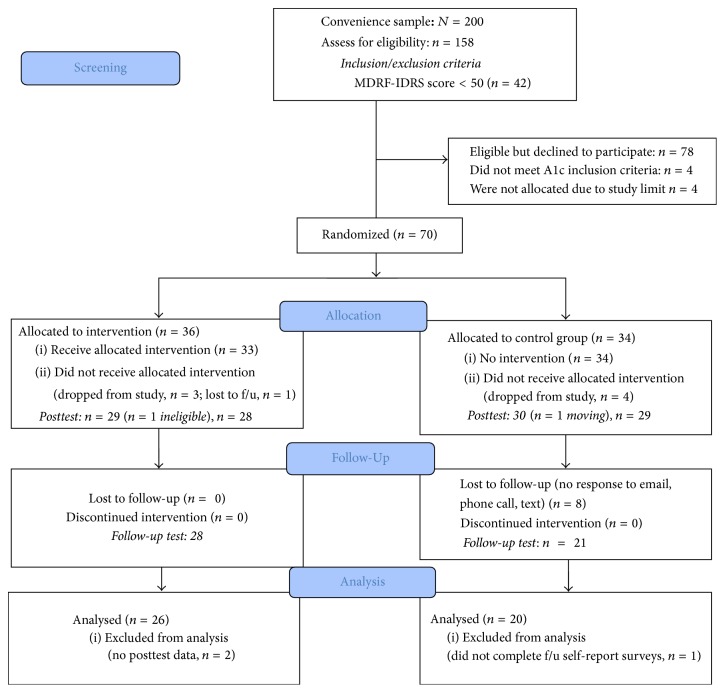
Diagram of flow of participants during study period.

An experimental, pretest-posttest control group repeated measures design was utilized for the intervention. Seventy eligible participants, aged ≥ 18 years, were randomized into either the 12-week group-based lifestyle intervention program or a control group that received standard print material on diabetes prevention. Baseline, postintervention (at 12 weeks), and follow-up (at 24 weeks) measures of body weight, waist circumference, blood pressure, glycated hemoglobin, self-reported physical activity levels, and self-reported health promoting dietary behaviors were obtained on all participants. The sample population was fairly homogenous since they were primarily* Gujarati* in origin with similar cultural, religious, and lifestyle practices. We utilized elements of the Community Based Participatory Research framework including raising awareness of diabetes risk factors during community health fairs, providing education on physical activity and eating habits during public health lectures, engaging with community members during festivals, and collaborating with the* mandir's* medical and executive committees about program goals, feasibility, timing, location, volunteer needs, and recruitment efforts for program implementation. The study was approved by the Institutional Review Boards at Rocky Mountain University of Health Professions and Texas Woman's University-Houston (see Figure 1 for diagram of flow of participants during study period).

### 2.2. Study Setting

The program was implemented at a Hindu temple or* mandir*, a place of worship, in Houston, Texas. The* mandir* collaborated with the research team to provide the physical space and equipment for the project. Dedicated* mandir *volunteers assisted with room set-up, weighing in participants, collecting weekly logs, distributing program materials, and providing oral translation in* Gujarati* of survey questions during data collection.

### 2.3. Selection of Subjects

A convenience sample of at-risk adult participants was selected from the* mandir*. Individuals were eligible if they were >18 years of age, had a diabetes risk score ≥ 50 as per the Madras Diabetes Research Foundation's Indian Diabetes Risk Score (MDRF-IDRS) [44], HbA1c value < 6.4% (≤47 mmol/mol), and willing to be randomized and complete all intervention assessments [45]. Participants were excluded if they (a) self-reported diagnosed diabetes, unstable chronic diseases (e.g., cardiac disease or cancer and/or were undergoing treatment), (b) were unable to participate in regular moderate intensity physical activity, (c) were pregnant (self-report) or planning a pregnancy in next 6 months, and/or (d) were currently involved in a supervised program for weight loss. Several screening events were held at the* mandir*. A print flyer was distributed to create awareness and encourage participation. Trained volunteers administered the MDRF-IDRS to 200 individuals in English or* Gujarati* and those who met the eligibility criteria (*n* = 158) were invited to take part in the study. Seventy-eight AIs consented to participate and completed baseline testing, including confirming eligibility via HbA1c testing. However, 4 were not eligible and 4 were not randomized due to meeting the maximum number of participants for the study.

### 2.4. Randomization

Seventy individuals were either randomized into a 12-week group-based lifestyle intervention program, a modified DPP program (*n* = 36), or a control group (*n* = 34) that received standard print material on diabetes prevention; participants were stratified by marital status to avoid contamination. To avoid contamination, intervention group participants were requested to not share or discuss session materials with other members in the congregation throughout the study period. The program was led by the principal investigator (PI), a trained expert who was blinded to all data collected. Each participant was assigned a numeric code that was used to label all data. The PI did not have access to the identifiable data until the completion of the study.

### 2.5. Intervention and Control Groups

All participants completed the pretest and attended a group orientation session before the intervention. Participants received a folder (control group) or 3-ring binder (intervention group) with their test results, their group allocation assignment, information about the study goals, posttest and follow-up test dates, dates/time/location for the 12-week program, and copies of weekly logs. Participants were encouraged to sign up for a text messaging app (https://www.Remind.com) so that they can receive weekly reminders from the research team about mailing in their weekly logs (control group) or coming to the weekly meeting with their logs (intervention group). A pedometer with instructions on its use and how to record daily step counts on the weekly logs was also provided to each participant. In addition, intervention group participants received resistance bands for exercise, a MyPlate plastic plate model, a set of measuring spoons and cups during the subsequent intervention sessions. Reminders to complete and mail in weekly logs were sent using the text messaging app and/or email. Participants were incentivized with a weekly $25 gift card drawing to a local grocery store for attending sessions and submitting the weekly logs.

### 2.6. 12-Week Lifestyle Intervention

The intervention targeted weight loss, increase in physical activity to 150 minutes/week or 10,000 steps per day, increase in fruit and vegetable intake (FVI) to a minimum of 5 servings per day, and decrease intake per day of foods with saturated and trans fats.

An evidence-based modified DPP curriculum, called the National Diabetes Education Program's (NDEP)* Power to Prevent (P2P): A Family Lifestyle Approach to Diabetes Prevention*, was used as the basis for the program. The* P2P *program was modified to be culturally tailored for Asian Indians. Individuals participated in weekly 75-minute group-based lifestyle intervention sessions held at the* mandir *on Sunday afternoons.

*P2P* is available for download from the following site: https://www.ndep.nih.gov/media/power-to-prevent-508.pdf?redirect=true. Participants were also taught how to set SMART (Specific, Measurable, Realistic, Achievable, and Time) goals choosing one small change they thought as achievable in their daily lives for the following week, keeping consistent with the theme of* Small Steps Big Rewards* that was part of the* P2P *program. Reinforcement and follow-up to the goals were done at the end of each session with a goal worksheet that was provided to self-select a behavior for change for the following week. At the beginning of the next session participants self-rated their achievement on a tracking sheet in their binder and discussed barriers/enablers to achieving their goals. Presentation notes and homework tasks were emailed to the group after each session. Additional information such as videos and recipes was also shared via email.

The control group received print materials available from the NDEP campaign* Small Steps Big Rewards Your GAME PLAN to Prevent Diabetes *[46]. The kit is available at https://ndep.nih.gov/publications/PublicationDetail.aspx?PubId=71. Each control group participant received a pedometer, 12 self-addressed stamped envelopes, and weekly logs (to record weight, steps walked, fruit/vegetable and fat intake) for each week of the intervention.

The session leader, a* Gujarati* American, facilitated each session and orally translated information to personalize it with examples of* Gujarati* colloquialisms, customs, and traditions. Specific barriers to adopting healthy behaviors were addressed through inspirational cultural messaging and visuals. Facilitator led 20 minutes of group physical activity time during 8 of the 12 sessions was provided to reinforce physical activity behavior change. Experiential methods such as exercise and cooking demonstrations, distribution of sample foods using healthier ingredients, a grocery store tour, and a recipe makeover pot-luck party were used to engage participants.

### 2.7. Data Collection and Study Measures

Outcomes were measured at baseline (preintervention), at 12 weeks (postintervention), and at 24 weeks (6-month follow-up) and all measurements took place at the* mandir *during the weekends. Primary clinical outcomes included weight (kg), hemoglobin A1c (mmol/mol), and waist circumference (cm). Six trained volunteers performed the measurements. Weight was measured using a standard scale (Detecto ProHealth D350 Dial Weight Scale), height was measured using a portable stadiometer (Seca 217 Stadiomenter), waist circumference was measured using a Gulik Anthropometric measuring tape following a standardized protocol of palpating the iliac crest, then taking a horizontal measurement just above it after the participant breathes out, and blood pressure was measured with the participant in the seated position using an automated blood pressure cuff (Healthsmart). The Bio-Rad Hemoglobin Capillary Collection System was used to obtain a blood sample via finger stick for the HbA1c test using the manufacturer's standardized protocol for specimen collection, labeling, storage, and shipping. Diabetes Diagnostic Laboratory (University of Missouri, Columbia) conducted the analysis for HbA1c. BMI was also calculated using Asian Indian BMI categories (normal > 23 kg/m^2^, overweight ≤ 23–24.9 kg/m^2^, obese ≥ 25 kg/m^2^). Intraobserver variation was minimized by providing volunteers with a data collection procedure manual with specific instructions and having them practice performing the measurements on each other and other volunteers prior to measuring the study participants.

Secondary outcomes were changes in physical activity and eating behaviors. The Health Promoting Lifestyle Profile II (HPLP II), a 52-item self-report questionnaire [47], was administered to assess intervention program goals. HPLP II was used to examine health promoting behaviors among* Gujarati* AI immigrants in the US demonstrating good reliability for the subscales (coefficient alphas: 0.76–0.84) and internal consistency of total scale = 0.94 [42].

### 2.8. Data Analysis

*A priori* power analysis was conducted using G^*∗*^Power version 3.1.6 to determine the minimum sample size required to find significance with a desired level of power set at .80, an *α*-level at 0.05, and a moderate effect size of *f* = 0.25 for the primary analysis. A minimum of 40 total participants were required to ensure adequate power for the Mixed Model Analysis of Variance (Mixed-Model ANOVA) [48–50]. Descriptive statistics and distributions of the continuous variables were examined for normality assumptions.

Baseline differences between groups were calculated using *t*-tests for continuous data (age, weight, BMI, HbA1c, waist circumference, blood pressure, HPLP II physical activity, and nutrition subscale mean score) and Chi-Square test for categorical data (gender, education, residency in US, English language fluency, occupation, marital status, MDRF-IDRS category, and type of diet). A mixed-model 2 × 3 ANOVA was used to analyze the mean differences between the 2 groups over 3 time points for the outcome variables (weight, HBA1c, and waist circumference) and also for changes in physical activity and fruit and vegetable intake. Alpha level was set at *p* < 0.05 and adjusted for simple effects. SPSS Statistics Version 23 (IBM, USA) was used for all data analysis.

## 3. Results

The* mandir* volunteers and the research team members distributed over 300 flyers during the weekly services and special events held at the* mandir*. Flyers were also posted around the* mandir* campus on the days of the screening events. Two hundred people came for the screening which took place over 3 different weekend days in October and November 2014 at the* mandir*. Individuals were screened using the MDRF-IDRS [44] and the American Diabetes Association (ADA) Risk test to determine eligibility as well as association with HbA1c. Of the 200 individuals screened, 42 were ineligible based on MDRF-IDRS screening tool (<50); thus, *n* = 158 were initially assessed as eligible. However, 78 declined to participate; 10 of the remaining 88 did not participate for baseline HbA1c testing due to a conflict with the scheduled dates or did not return phone calls or emails. Hence, we were able to schedule 78 Asian Indians for baseline hemoglobin A1c (HbA1c) testing to determine eligibility for inclusion criteria. For the sample of 78 tested with HbA1c, findings indicated that the MDRF-IDRS significantly predicted HbA1c (*R*^2^ = 0.101, *p* = 0.005) versus the ADA Risk test which did not (*p* = 0.139), thus providing validation for use of the MDRS-IDRS screening tool for our study sample. Of the 78 tested, *n* = 4 did not meet inclusion criteria for HbA1c. Of the remaining participants, 70/74 were randomly allocated to the intervention group (*n* = 36) or control group (*n* = 34). Four people were not allocated because the study was approved for a maximum of 70 participants. All participants that consented to and completed baseline testing (*n* = 78) were invited to a meeting at the* mandir* to receive their results and allocation assignment. For the 4 that were deemed ineligible, a* mandir* volunteer physician was available to discuss their results and receive advice regarding further medical testing to determine diabetes diagnosis.

The intervention program was conducted for 12 consecutive weeks from December 2014 to February 2015 on Sunday afternoons at the* mandir*. Average weekly attendance was 22 participants for the duration of the program. On average, intervention group participants attended 7.4/12 sessions. Retention rate at posttest (12 weeks) was 80% for the intervention group and 83% for the control group. The dropouts were similar in demographics to baseline cohort at pretest. For the follow-up test (24 weeks), the retention rate dropped to 72.2% for the intervention group and 58.82% for the control group. Again, demographics of those that dropped out were similar to those in the sample at pretest and posttest. Identified factors for dropping from the program included lack of time and family obligation, moving out of Houston, ineligibility after posttest due to increase in HbA1c, or having a schedule conflict and inability to come on the dates available for posttest and/or follow-up.

The mean age of the 70 participants was 53.26 ± 11.49 years; the majority (54.3%) were females, were married (94.3%), reported English as their 2nd language (62.9%), lived in the US for more than 8 years (82.9%), and were college educated (77.2%). Two-thirds or 64.3% of the participants reported they worked full-time, and 75.7% followed a lactovegetarian diet. Mean HbA1c was 38.41 mmol/mol (SD ± 3.8); mean waist circumference for males and females was 88.07 cm (SD ± 11.03) and 78.92 cm (SD ± 8.33), respectively; mean systolic and diastolic BP were 123.96 mmHg (SD ± 2.15), and 79.57 mmHg (SD ± 1.07), respectively. In the study sample (*n* = 70), no significant differences between the intervention and control group were noted at baseline for demographic characteristics of age, gender, number of years of residency in US, English language fluency, education, diet, marital status, and occupation (see Table 1). This was also true for the complete cases (*n* = 46) that were analyzed (see Table 2). Furthermore, the two groups within the complete cases analyzed were similar in their baseline clinical variables (weight, BMI, waist circumference, HbA1c, and blood pressure) and self-reported diet and physical activity scores (see Table 3).

### 3.1. Primary Outcomes

For weight loss, we found a significant main effect of time regardless of group at *F*(1.53, 67.29) = 33.57, *p* < 0.0005. Follow-up analysis revealed significant differences between baseline and posttest and baseline and follow-up at *p* < 0.0005 for both. In terms of weight, 84.8% of our sample was overweight or obese at baseline per Asian Indian BMI cut-offs. Over the course of the intervention from baseline to follow-up at 24 weeks, 35/46 (76.1%) of the participants lost 5% or more of their total body weight, which approached significance (*p* = 0.052). For BMI, we found a significant main effect of time regardless of group at *F*(1.55, 68.2) = 34.964, *p* < 0.0005. Follow-up analysis revealed significant differences between baseline and posttest and between baseline and follow-up at *p* < 0.0005 for both. However, HbA1c changes showed a significant interaction of time and group at *F*(2, 88) = 17.116, *p* < 0.0005 with follow-up analysis of simple effects indicating significant differences in the intervention group between pre and post and pre and follow-up (*p* < 0.0005). In terms of HbA1c, 45.6% (21/46) were classified as having prediabetes. Over the course of the intervention, from baseline to follow-up at 24 weeks, 26.9% of the intervention group participants had a reversal from prediabetes to normoglycemia compared to the control group that had an inverse change, with 5% of the group progressing from normoglycemia to prediabetes. Changes to abdominal obesity or waist circumference were similar with a significant interaction of time and group at *F*(2, 88) = 3.337, *p* = 0.04. Follow-up analysis of simple effects revealed a significant difference in the intervention group between baseline and 12 weeks (*p* = 0.015) (see Table 4).

### 3.2. Secondary Outcomes

The goals of the intervention program were to increase the participants' physical activity levels and fruit and vegetable intake as well as lose weight. Based on the mean scores on the HPLP II Physical Activity subscale, there was a significant main effect of time regardless of group at *F*(2, 84) = 11.512, *p* < 0.0005 with follow-up analysis showing significant differences between baseline and posttest at 12 weeks (*p* < 0.0005) and baseline and follow-up test at 24 weeks (*p* = 0.001). Also, based on the mean scores on the HPLP II Nutrition subscale, there was a significant main effect of time regardless of group at *F*(2, 84) = 10.086, *p* < 0.0005. However, positive changes in dietary habits were noted among intervention group participants as compared to the control group individuals; the mean score increase was significant from 0 and 12 weeks (*p* = 0.002) and 0 and 24 weeks (*p* < 0.0005) in the intervention group only (see Table 2).

## 4. Discussion

This is the first randomized control trial to effectively engage a local Hindu* mandir *and use modified DPP materials from the NDEP's* P2P* program to tailor a lifestyle intervention for a* Gujarati* Asian Indian community in the US. The main purpose was to show that successful translation of a 12-week culturally tailored group-based lifestyle intervention, facilitated by a trained bilingual healthcare professional, was more effective than general advice or print materials to help participants reduce HbA1c, a main clinical risk factor for developing diabetes.

Our attendance rate was similar to that reported in other DPP translational studies carried out in faith-based settings [38, 51, 52] such as African American churches, though the program length varied from 6 to 16 sessions in those studies. Of the 2 recent DPP translational studies that targeted US South Asian communities, neither took place in a faith-based setting [39, 40]. The randomized SAHELI trial [40] took place in a medically underserved neighborhood of South Asian immigrants in Chicago with the average attendance reported to be 5/6 sessions or 83%. The nonrandomized RICE study [39] took place in 2 predominantly* Sikh* Asian Indian neighborhoods in NYC with one neighborhood allocated to the treatment arm and the other neighborhood allocated to the control group. In this study, 97% of the intervention group participants attended 4/6 or 66.6% of the sessions. Our intervention cohort was primarily* Gujarati* Asian Indians that frequented a* mandir* in the Houston area. Our average attendance was 7.4/12 or 61.6%, with a majority (55.5%) of the intervention group participants attending 9 to 12 sessions. Longer duration (12 sessions versus 6 sessions), as well as the timing of our program (December–February), may have impacted our attendance rate.

For retention, we utilized a text messaging app (https://www.remind.com) and email communication weekly during the intervention phase. Though voluntary, we had 24/36 intervention group participants and 24/34 control group participants that signed up to receive weekly text messaging. In addition, phone calls were made to schedule and remind all participants to come for data collection. Due to limited resources, we were not able to provide follow-up sessions during the postintervention phase which can improve retention. For example, in the RICE study [39], trained community health workers (CHWs) conducted up to 10 follow-up phone calls as well as face-to-face meetings to collect data with a retention rate of 85.7% as compared to 65.7% at 24 weeks for our study. Similarly, in the SAHELI trial [40], retention rate was reported as 100% stating that “no participant was lost to follow-up” although the authors utilized intention to treat for data analysis at 24 weeks after intervention. Follow-up phone calls and community* melas* or health fairs were used to reinforce intervention strategies from the group sessions, though it was reported that only 16% of the intervention group participants completed at least 3/10 phone counseling sessions and participation in the* melas* dropped to 12% by the 4th and final* mela*. Other barriers for retention included (1) limited dates and times for data collection due to space unavailability at the* mandir*, (2) limited availability of the volunteer data collectors, and (3) inability of participants to come for all data collection points due to family, travel, or work obligations. Hence, data collection was scheduled at a convenient place (*mandir*), time/day (weekends), and participants received $10 cash incentive. Future studies should explore if using CHWs, providing more options for follow-ups, more opportunities for data collection, and different incentives, would improve retention rates.

Weight loss occurred in both the intervention and control group participants; however, no significant differences were noted between the intervention and control group at 12 or 24 weeks. This may have been due to increased accountability for all participants with the requirement to document daily weight along with physical activity and fruit and vegetable intake on their weekly logs. Previous US DPP translational studies [38, 51, 52] conducted in faith based settings have reported weight loss over time, though some of these studies did not include a control group. In a recent community trial [53] of faith-based lifestyle intervention to prevent diabetes among African Americans, the authors reported that intervention group participants lost significantly more weight than those in the control group and that attendance to weekly program sessions by intervention group members modified the effect of weight loss. In the RICE study [39], significant weight loss was reported within the treatment group but between group differences for changes in weight was not significant. The RICE study design did not have random group assignment and there were significant baseline differences between groups which may have reduced the efficacy of the findings. In the SAHELI trial [40], the randomized intervention group exhibited a significant weight loss compared to the control group. Also, the study participants had much higher BMI (mean 29 kg/m^2^) versus 26.32 kg/m^2^in our study, and 15.2% of our sample was normal weight at baseline. Yet, a greater weight loss was achieved at 24 weeks after intervention in our study (i.e., 34.6% of the intervention group participants achieved a 5% weight loss) as compared to the SAHELI trial (19% at 24 weeks).

A behavioral strategy that we employed was to have the intervention group participants “weigh in” at the beginning of each session to foster external accountability which has been shown to promote weight loss [54]. Furthermore, similar to Sattin and colleagues [53], we also noted a positive trend between the number of sessions attended and weight loss. These positive weight changes by participants further alludes to the effectiveness of the culturally tailored intervention program to induce weight loss among Asian Indians.

The intervention was successful in lowering blood glucose levels or HbA1c with the intervention group participants, lowering their HbA1c significantly more than the control group. The number of participants with prediabetes decreased in the intervention group from 57.6% at baseline to 30.8% at 6-month follow-up; 69.2% of intervention group participants were in the normal HbA1c range of ≤5.4 at 6-month follow-up as compared to 42.3% at baseline. In contrast, in the control group, the number of participants with normal HbA1c decreased from 70% at baseline to 65% at 6-month follow-up and the number of participants who had prediabetes per HbA1c increased from 30% at baseline to 35% at 6-month follow-up. HbA1c is a clinical measure of risk for diabetes and these results indicate that the intervention group participants not only reduced their risk for diabetes by shifting from prediabetes to normoglycemia at the end of the intervention but continued to maintain and lower glucose levels at 6 months, despite no follow-up between 3 and 6 months of the program. This further attests to the effectiveness of a modified DPP intervention for at-risk* Gujarati* Asian Indians in this study and concurs with prior studies that used either HbA1c [40] or 2-hour fasting glucose to assess prediabetes status of participants [39]. None of the other DPP based translational studies [38, 51] within community settings utilized HbA1c, thus making comparisons difficult.

The intervention was successful in decreasing waist circumference from baseline to posttest (12 weeks) within the intervention group but not within the control group. Similar results were reported with significant changes within the intervention group but not within the control group or between groups in the SAHELI trial [40]. In the RICE study [39], significant decrease in waist circumference was reported within both groups but not baseline to 24-week follow-up. Literature suggests that more than body weight, waist circumference is strongly associated with cardiometabolic risk in South Asians [17, 55–57]. Our results support this association since the intervention showed a significant decrease between groups in waist circumference but not in body weight. Literature also suggests that besides body weight, and waist circumference, visceral adiposity is strongly associated with insulin resistance and diabetes among Asian Indian [16, 58]. Therefore, future studies could measure body fat percentage and observe the association of overall body fat percentage to changes in a clinical variable such as HbA1c or Oral Glucose Tolerance Test (OGTT).

Regular moderately intense physical activity is associated with a substantially lower risk of diabetes [59]. Yet consistently lower levels of physical activity have been reported in studies conducted on US Asian Indians [21, 42, 60]. Use of the physical activity subscale of the HPLPII, a self-report measure that has been validated on US* Gujarati* Asian Indians [42] is a strength of our study. Our participants' mean score on the physical activity subscale improved between 0, 3, and 6 months, regardless of group assignment. This positive change may have been due to the fact that pedometers issued to all participants provided the motivation and reinforcement to increase physical activity along with the accountability of documenting steps on the weekly logs. Physical activity has been assessed using different measures among Asian Indians, thus making comparisons among studies difficult. For example, the RICE study [39] utilized a single dichotomous self-reported question to assess physical activity; however, this does not address frequency, intensity, time, and type of physical activity. In the MASALA study [61], the Typical Week's Physical Activity Questionnaire was used [62], though no information was provided about the validation of this instrument in Asian Indians. Further development of a brief self-report tool that accurately reflects the frequency, intensity, time, and type of physical activity is necessary to determine which aspects of physical activity have the most benefit in terms of diabetes risk among Asian Indians in the US.

Among immigrant Asian Indians in the US, FVI intake varies by their region of origin in India. One study that compared food intake of AIs originally from North, South, and West India showed fruit consumption for participants originally from West India (states of Gujarat and Maharashtra) was significantly lower (*p* < 0.05) than for those from North India (states of Punjab, Rajasthan, and Uttar Pradesh) [63]. Baseline FVI, as assessed by 2 questions from the HPLPII instrument, showed that* Gujarati *participants in this study had similar results; with 43.5% reporting that they never or sometimes eat 2–4 servings of fruit per day and 41.3% reporting that they never or sometimes eat 3–5 servings of vegetables per day. Another study indicated that US* Gujarati* Asian Indians need to be educated about the USDA recommendations for servings per day from each food group and about how to incorporate traditional Indian foods into the daily recommendations for FVI for overall healthy eating [64]. Thus, our intervention emphasized serving sizes, appropriate portions, and using MyPlate to create balanced vegetarian and* Gujarati* meals since lower levels of FVI have been postulated to account for some of the disparity in diabetes prevalence among Asian Indians in the US [65]. We utilized the validated nutrition subscale of the HPLP II instrument to track changes in eating habits among participants. The intervention was successful in improving dietary behavior among participants, regardless of group assignment. This positive change may have been due to improved knowledge on portion sizes and serving sizes provided in the print materials to all participants. The control group also received the NDEP Small Steps Big Rewards Your GAME PLAN to Prevent Diabetes kit [46] which contains a fat and calorie counter and a food and activity tracker. Food trackers and diaries have shown to increase awareness of eating habits and reinforce serving sizes and portion control though an easy to use online food tracker or app may increase compliance of submitting this information in future studies.

### 4.1. Strengths and Limitations

Strengths of our study were as follows: We engaged the community and got buy-in from community leaders from the beginning; we utilized an experimental randomized control group design with a homogenous sample of* Gujarati* Americans; and we were able to implement the program in a convenient real world faith-based community setting (a Hindu* mandir*), thus increasing the external validity of our findings to* Gujarati* Americans elsewhere. Also, our groups were similar at baseline; thus our results are more likely due to true differences between groups; we utilized a text messaging app and email to minimize attrition and to schedule post and f/u data collection appointments; the* mandir* infrastructure, space, and volunteer support were crucial to the study's success; our program facilitator was a trained bilingual* Gujarati* American; and our translational DPP program was based on an existing evidence based program modified using current literature to suit the needs of* Gujarati *Asian Indians. We also faced several limitations. Our study was conducted at only one location; we did not get objective measurement of physical activity; and due to limited resources, we did not translate print and presentation materials into* Gujarati* or utilize other means of tracking fruit and vegetable intake that could have increased eating behavior change. Future studies should investigate offering the modified 12-week program to a heterogeneous cohort of South Asians (beyond* Gujaratis*) to increase generalizability of findings. Limited resources also prohibited us from providing follow-up sessions for our intervention group, and space and scheduling constraints at the* mandir* limited the time frame for the intervention which accounted for some attrition. Another limitation was that sustainability of the intervention program was not addressed directly as part of this study. In the future, the* mandir's* leadership and the research team plan to explore sustainability and scalability issues including use of trained CHWs or health coaches to provide the intervention.

## 5. Conclusions

Findings from this study demonstrated that a culturally tailored, group-based lifestyle intervention program provided in a faith-based community setting can effectively reduce HbA1c, a main risk factor for T2DM, among* Gujarati* Asian Indians living in the US. To our knowledge this is the first randomized control trial of a translational DPP program targeted towards the* Gujarati* community in the US and the first one offered at a Hindu* mandir *in the US. This study helps answer important initial questions regarding the feasibility, acceptability, and effectiveness of a 12-week culturally tailored program in such a setting. Findings from this initial study will serve as a basis to further refine data collection tools and methods for clinical and behavioral variables including feasibility of oral interviews or bilingual questionnaires for use in real world settings. Future studies could assess how stage of health behavior change, self-efficacy, and social support may influence outcomes that could lead to reduction in risk factors for diabetes among Asian Indians in the US.

It will also be important to identify factors that contribute to successful implementation of DPPs in real-world community settings. The adaptability and scalability of this 12-week program to a broader South Asian population should be explored further using a metric such as the PIPE Impact framework [66, 67] that can systematically help address the knowledge gap that still exists in identifying factors for conducting translational DPP programs in community settings. This information is necessary in order to determine which translational DPP programs will have the most impact at the population level. Development, implementation, and evaluation of effective translational DPP programs for South Asian communities are a priority as this demographic continues to grow. Community engagement in prevention programs can help shift the rising tide of diabetes among South Asians in the US.

## Figures and Tables

**Table 1:** Baseline demographic characteristics of study sample by group (*N* = 70).

Characteristic	Intervention group	Control group	*p* value
	(*n* = 36)	(*n* = 34)	
	*N* (SD, %)	*N* (SD, %)	
*Age in years, mean (SD)*	53 (11.0)	53.6 (12.2)	0.436^**∗**^
*Gender*			
Male	16 (44.4)	16 (47.1)	0.826^**∗∗**^
Female	20 (55.6)	18 (52.9)	
*Years in US*			
Born in US	0 (0.0)	1 (2.9)	0.304^**∗∗**^
Immigrant: less than 8 years	4 (11.1)	7 (20.6)	
Immigrant: more than 8 years	32 (88.9)	26 (76.5)	
*Language fluency*			
English as primary language	6 (16.7)	8 (22.2)	0.501^**∗∗**^
English as second language	25 (69.4)	19 (52.8)	
Not fluent in English	5 (13.9)	7 (19.4)	
*Education*			
High school	3 (8.3)	10 (27.8)	0.077^**∗∗**^
Technical/associate's degree	1 (2.8)	2 (5.6)	
Bachelor's degree	16 (44.4)	14 (38.9)	
Graduate degree	16 (44.4)	8 (23.5)	
*Diet*			
Vegan	9 (25.0)	5 (13.9)	0.452^**∗∗**^
Lactovegetarian	25 (69.4)	28 (77.8)	
Ovolacto vegetarian	2 (5.6)	1 (2.8)	
Nonvegetarian	0 (0.0)	0 (0.0)	
*MDRF-IDRS*			
Medium risk category	12 (33.3)	9 (26.4)	0.531^**∗∗**^
High risk category	24 (66.7)	25 (73.6)	
*Marital status*			
Married	33 (91.7)	33 (97.1)	0.331^**∗∗**^
Widowed	3 (8.3)	1 (2.9)	
*Occupation*			
Work full-time	23 (63.9)	22 (61.1)	0.463^**∗∗**^
Work part-time	1 (2.8)	4 (11.8)	
Retired	4 (11.1)	5 (13.9)	
Go to school	1 (2.8)	0 (0.0)	
Home maker	4 (11.1)	2 (5.6)	

^*∗*^Independent  *t*-test, ^*∗∗*^chi-square test.

**Table 2:** Baseline demographic characteristics of complete cases analyzed by group (*N* = 46).

Characteristic	Intervention group	Control group	*p* value
	(*n* = 36)	(*n* = 34)	
	*N* (SD, %)	*N* (SD, %)	
*Age in years, mean (SD)*	52.7 (9.8)	53.6 (12.5)	0.813^**∗**^
*Gender*			
Male	12 (46.2)	11 (55.0)	
Female	14 (53.8)	9 (45.0)	
*Years in US*			
Born in US	0 (0.0)	1 (5.0)	0.358^**∗∗**^
Immigrant: less than 8 years	2 (7.7)	3 (15.0)	
Immigrant: more than 8 years	24 (92.3)	16 (80.0)	
*Language fluency*			
English as primary language	3 (11.5)	5 (25.0)	0.501^**∗∗**^
English as second language	21 (80.8)	14 (70.0)	
Not fluent in English	2 (7.7)	1 (5.0)	
*Education*			
High school	1 (3.8)	5 (25.0)	0.083^**∗∗**^
Bachelor's degree	12 (46.2)	9 (45.0)	
Graduate degree	13 (50.0)	6 (30.0)	
*Diet*			
Vegan	5 (19.2)	4 (20.0)	0.675^**∗∗**^
Lactovegetarian	20 (76.9)	16 (80.0)	
Ovolacto vegetarian	1 (3.8)	0 (0.0)	
Nonvegetarian	0 (0.0)	0 (0.0)	
*MDRF-IDRS*			
Medium risk category	7 (26.9)	6 (30.0)	0.818^**∗∗**^
High risk category	19 (73.1)	14 (70.0)	
*Marital status*			
Married	24 (26.9)	19 (95.0)	0.714^**∗∗**^
Widowed	2 (73.1)	1 (5.0)	
*Occupation*			
Work full-time	18 (69.2)	15 (75.0)	0.725^**∗∗**^
Work part-time	1 (3.8)	2 (10.0)	
Retired	3 (1.5)	2 (10.0)	
Go to school	1 (3.8)	0 (0.0)	
Home maker	3 (11.5)	1 (5.5)	

^*∗*^Independent  *t*-test, ^*∗∗*^chi-square test.

**Table 3:** Baseline measures of complete cases analyzed by group (*N* = 46).

Variable	Intervention group		Control group		*p* value
	(*n* = 26)		(*n* = 20)		
*Weight (kg), mean (SD)*	67.4	(11.6)	65.5	(10.1)	0.570^*∗*^
*BMI (kg/m* ^*2*^ *), mean (SD)*	26.32	(3.5)	25.66	(3.6)	0.539^*∗*^
*BMI category (kg/m* ^*2*^ *), n (%) *					0.464^*∗∗*^
<23	4	(15.4)	3	(15.0)	
≥23 to <25	5	(19.2)	7	(35.0)	
≥25	17	(65.4)	10	(50.0)	
*Waist circumference (cm), mean (SD) *	83.6	(10.5)	83.1	(11.1)	0.862^*∗*^
*Men, number (%)*					0.114^*∗∗*^
≤85 cm	2	(16.7)	6	(54.55)	
≥85 cm	10	(83.3)	5	(45.45)	
*Women, number (%)*					0.149^*∗∗*^
≤80 cm	8	(57.14)	6	(66.67)	
≥80 cm	6	(42.86)	3	(33.33)	
*Hemoglobin A1c (mmol/mL), mean (SD)*	39	(3.8)	37.7	(3.7)	0.251^*∗*^
Hemoglobin A1c (%), mean (SD)	5.7	(0.37)	5.6	(0.34)	0.308^*∗*^
*Diabetes classification, n (%)*					0.062^*∗∗*^
<39 mmol/mol	11	(42.3)	14	(70.0)	
≥39 to <48 mmol/mol	15	(57.7)	6	(30.0)	
*Blood pressure (mmHg), mean (SD) *					
Systolic blood pressure	119	(12.6)	126.3	(16.6)	0.097^*∗*^
Diastolic blood pressure	77.5	(7.7)	126.3	(8.8)	0.237^*∗*^
*Physical activity and nutrition: mean (SD)*					
HPLP II physical activity subscale	2.11	(0.86)	2.11	(0.69)	0.931^*∗*^
HPLP II nutrition subscale	2.67	(0.46)	2.92	(0.41)	0.065^*∗*^

^*∗*^Independent  *t*-test, ^*∗∗*^chi-square test.

**Table 4:** Changes in outcomes, baseline, posttest, and follow-up complete cases analyzed by group (*N* = 46).

Outcome	Intervention	Control
	Mean (SD)	Mean (SD)
Primary outcomes	*n* = 26	*n* = 20
*Weight *(*kg*)^a,b,c^		
Baseline	67.41 (11.56)	65.54 (10.11)
Posttest	64.90 (11.71)	64.12 (10.04)
Follow-up	64.86 (11.40)	64.18 (9.20)
*HbA1c *(*mmol*/*mol*)^b,c,d^		
Baseline	38.96 (3.73)	37.65 (0.34)
Posttest	37.00 (3.53)	37.85 (3.82)
Follow-up	36.54 (3.95)	37.75 (3.61)
*Waist circumference *(*cm*)^b,d^		
Baseline	83.64 (10.49)	83.08 (11.07)
Posttest	82.15 (11.28)	82.60 (10.85)
Follow-up	82.19 (11.67)	84.15 (11.03)
Secondary outcomes:		
*HPLP II Physical Activity Subscale* ^a,b,c^		
Baseline	2.11 (0.86)	2.12 (0.73)
Posttest	2.62 (0.76)	2.47 (0.63)
Follow-up	2.62 (0.74)	2.39 (0.67)
*HPLP II Nutrition Subscale* ^a,b,c^		
Baseline	2.67 (0.46)	2.94 (0.42)
Posttest	3.00 (0.32)	3.07 (0.39)
Follow-up	3.04 (0.34)	3.10 (0.43)

^a^Significant main effect of time regardless of group: weight: *p* < 0.0005, HPLP II physical activity: *p* < 0.0005, HPLP II nutrition: *p* < 0.0005.

^b^Significant differences between baseline versus posttest: weight: *p* < 0.0005, HbA1c: *p* < 0.0005, waist circumference: *p* = 0.015, HPLP II physical activity: *p* < 0.0005, HPLP II nutrition: *p* = 0.002.

^c^Significant differences between baseline versus follow- up: weight: *p* < 0.0005, HbA1c: *p* < 0.0005, HPLP II physical activity: *p* < 0.0005, HPLP II nutrition: *p* < 0.0005.

^d^Significant time × group interaction: HbA1c: *p* < 0.0005, waist circumference: *p* = 0.04.
